# A survivorship-oriented enhanced care model for patients undergoing radical prostatectomy

**DOI:** 10.1371/journal.pone.0346609

**Published:** 2026-04-03

**Authors:** Xiuqun Yuan, Yuting Chen, Huihui Lu, Pei Zheng, Yanyan Zhang, Min Chen, Xia Sheng

**Affiliations:** 1 Department of Nursing, Renji Hospital, School of Medicine, Shanghai Jiao Tong University, Shanghai, China; 2 Department of Nursing, Shanghai Fourth People’s Hospital, School of Medicine, Tongji University, Shanghai, China; 3 Department of Health Management Center, Shanghai Fourth People’s Hospital, School of Medicine, Tongji University, Shanghai, China; Jan Biziel University Hospital No 2 in Bydgoszcz: Szpital Uniwersytecki Nr 2 im dr Jana Biziela w Bydgoszczy, POLAND

## Abstract

**Objectives:**

Post-prostatectomy patients experience urinary incontinence, fluctuating quality of life, and psychosocial distress during early survivorship. Evidence-based nursing models addressing long-term supportive needs remain limited. This study developed and evaluated an enhanced survivorship-oriented care model designed to improve postoperative functional recovery and quality-of-life outcomes.

**Methods:**

A retrospective study was conducted at a tertiary urologic center. The improved survivorship model was developed based on our previous PROSTATE care model, integrating nurse-led continuous follow-up, psychosocial support, and structured rehabilitation. A total of 1062 patients who underwent radical prostatectomy between June 2024 and May 2025 received the enhanced survivorship care, compared with 673 patients treated between June 2023 and December 2023 under the previous PROSTATE care model. Outcomes included urinary continence, quality of life, postoperative complications, and length of stay. Between-group comparisons were performed using independent-samples tests, and repeated-measures ANOVA was applied to assess longitudinal changes.

**Results:**

Both groups demonstrated significant improvements in urinary recovery and quality of life over time (time × group interaction, P < 0.005). The enhanced-care group showed superior functional recovery, with lower ICIQ-SF scores at catheter removal and 3 months (both P < 0.005), and consistently higher FACT-P scores across all follow-up time points (all P < 0.005), with a large effect size at 3 months (Cohen’s d = 0.89). Length of stay was reduced without an increase in overall complication rates. The incidence of urinary fistula was significantly lower in the enhanced-care group (0.76% vs. 2.75%; RR = 0.28, 95% CI 0.12–0.64, P = 0.001).

**Conclusion:**

The enhanced survivorship care model demonstrated clinically meaningful improvements in quality of life and continence recovery, while maintaining patient safety. These findings support its clinical value and potential for wider implementation as a structured survivorship strategy following radical prostatectomy.

## Introduction

Prostate cancer (PCa) remained the first in male adults with estimated 299010 new cases worldwide [1], and the incidence raised rapidly in China these years [2]. The benchmark treatment for patients with localized prostate cancer is radical prostatectomy, either via open, laparoscopic or robot-assisted approach [3]. However, patients might suffer urinary incontinence, erectile dysfunction, and other treatment-related adverse events after whatever ways of surgeries, which substantially impacted their functional recovery and quality of life (QOL) [4]. Although enhanced recovery after surgery (ERAS) programs have been widely adopted in urological practice, these approaches primarily target perioperative safety and short-term outcomes rather than long-term survivorship [5].

According to urologic oncology survivorship guidelines, prostate cancer (PCa) survivorship care should encompass functional rehabilitation, psychosocial adaptation, management of treatment-related side effects, and long-term health promotion [6,7]. However, evidence-based nursing models specifically targeting the early survivorship care after prostatectomy remain limited. Existing nurse-led supportive interventions primarily focused on managing postoperative symptoms like urinary incontinence and demonstrated benefits in reducing psychological distress, improving early continence and facilitating early cancer adjustment [5,8]. However, these interventions lacked continuity, interdisciplinary coordination, and long-term monitoring that extends beyond the immediate postoperative period.

Our team previously established the “PROSTATE” perioperative care model [9] to optimize in-hospital recovery and streamline multidisciplinary collaboration for patients underwent radical prostatectomy. However, this model did not adequately address patients’ ongoing supportive needs after discharge. Complementary to these findings, our qualitative research among prostate cancer survivors revealed persistent fears of cancer recurrence, insufficient access to continuous professional guidance, and unresolved concerns related to sexual dysfunction like limited support for intimacy alternation and masculinity-related concerns. Above findings underscore the necessity to transition from a perioperative-focused care framework to a more comprehensive survivorship-oriented model that integrates physical, emotional, informational, and sexual health support throughout early recovery. Building on this rationale, the survivorship care framework advocates holistic and continuous support that extend care beyond the perioperative period.

The survivorship care framework emphasizes holistic, continuous support that extends beyond acute treatment and into long-term recovery. Given the increasing need for structured long-term support among prostate cancer survivors, we developed an enhanced nurse-led care model grounded in the survivorship care framework. The model integrates an andrologist and a full-time prostate cancer nurse specialist to provide coordinated functional rehabilitation, psychosocial support, and life-long monitoring after radical prostatectomy. This study aimed to evaluate the feasibility and effectiveness of this enhanced care model in improving postoperative urinary outcomes, psychosocial adjustment, and overall quality of life, compared with conventional care.

## Materials and methods

### Study population

The study was approved by the Institutional Review Board of our hospital (Ethical Approval No. KY2018−212, SJUPN-HY-202312–23-KS1). Participants in the study understood the details of the research and have given the written informed consent.

This retrospective study compared 1062 patients who underwent radical prostatectomy between June 2024 and May 2025 with 673 patients who underwent the procedure between June 2023 and December 2023. Data were first accessed for research purpose in 01/06/2025. All participants were adult male patients treated at our hospital by subspecialized urological surgeons in prostate cancer. Patients with severe comorbidities, such as end-stage renal disease, heart failure, or other systemic diseases, were excluded. The study was designed as a comparative analysis between the survivorship care model and our previous PROSTATE care model to evaluate the impact of the enhanced nursing interventions on postoperative outcomes after radical prostatectomy.

### Development of the improved survivorship care model based on the PROSTATE care model

The reconstruction of the survivorship care model followed a systematic, multi-phase development process grounded in the existing PROSTATE care framework, current survivorship guidelines, and the empirical needs of prostate cancer survivors. The first phase involved an evidence synthesis focusing on functional recovery, psychosocial adjustment, and long-term supportive care after radical prostatectomy, which informed the preliminary structure and key components of the enhanced model. The second phase consisted of a qualitative needs assessment to ensure that the proposed interventions aligned with the preference of patients, caregivers, surgeons, and allied health professionals. Semi-structured interviews were conducted using an interview guide (Table 1) developed from the initial literature review. Purposive sampling was used to ensure variation in age, disease stage, pathology, treatment modality, and socioeconomic background. This approach ensured a comprehensive understanding of unmet needs and barriers across different subgroups. The third was integrate above results to draft the survivorship care model. A two-round expert consultation was then conducted with urologists, andrologist, urological nurse specialists, rehabilitation specialists, and psycholgoical related health professions. Experts evaluated the model for clarity, feasibility, clinical relevance, and completeness of intervention components. Revisions were made based on consensus and applicability in the hospital setting. Lastly was a pilot study to assess patient acceptance and feedback from all stakeholders. The final survivorship care model was established when no further revisions were needed.

**Table 1:** The interview outline of related stakeholders.

Stakeholders	Main questions
Administrators (chief nurse officer, nurse managers)	From your perspective, what key factors are essential to support the successful implementation of this survivorship care model? What organizational facilitators or resources are needed to sustain the model in routine practice? For example, staffing, training, or workflow integration. How to monitor and evaluate the quality of implementation within our hospital context? What other challenges or difficulties will you anticipate to meet at the institutional or system level, and how might they be addressed?
Surgeons(Urologists/Andrologists)	What you think is important to improve patient outcomes before and after the radical prostatectomy? What challenges do you observe in maintaining patients’ long-term adherence to postoperative rehabilitation and follow-up? What kinds of nurse-led multidisciplinary support do you believe are most beneficial for improving long-term recovery? How can we better integrate the survivorship care model into the routine workflow?
Patients (Prostate Cancer Survivors)	After being diagnosed with prostate cancer, what were your primary concerns or unmet needs? Or what kind of support do you need? After radical prostatectomy, what challenges did you experience in daily life, recovery, or psychosocial adjustment? What forms of professional support (e.g., education, follow-up, symptom management, psychological assistance) would help you the most during recovery? What do you expect from healthcare providers regarding long-term survivorship support?

The improved survivorship care model added the following four elements:

a. *Continuous nurse-led follow-up and monitoring:* The nurse specialist served as the primary coordinator between urologists, andrologists, wound nurses, and rehabilitation specialists to ensure transition across care settings. Surgical preparation and long-term follow-up was conducted to collect data including treatment-related symptoms, postoperative Prostate-Specific Antigen (PSA) surveillance, and early identification of other health-related comorbidity. Additional online consultations and disease-related information were provided at their first clinic visit after they were diagnosed to reduce uncertainty. For patients with a family history of prostate cancer or male first-degree relatives (such as sons or brothers), the nurse specialist offered individualized counseling and recommended early PSA screening for timely detection. All related educational materials were available in both written and digital formats to reinforce learning and promote long-term self-management.
b. *Management of fear of cancer recurrence*: Literature and our qualitative results all found the fear of recurrence was the most annoying issues for patients after radial prostatectomy [10]. Targeted psychological support was incorporated to relieve patients’ worries and anxiety. The nurse specialist screened for psychological problems through the Hospital Anxiety and Depression Scales (HADS) each time when patients came to the hospital for follow-up, and delivered brief coping strategies through materials developed by our research team. Patients with persistent high HADS scores were referred to psychosocial specialists through a fast referral pathway.
c. *Enhanced symptom management*: Urinary incontinence was the most distressing postoperative symptom, often affecting patients’ family dynamics and social participation [11]. After catheter removal, patients were referred to a rehabilitation nurse for structured pelvic floor muscle training (PFMT). The rehabilitation nurse conducted weekly telephone follow-ups to monitor continence recovery and ensure correct PFMT techniques. For patients who failed to achieve obvious improvement despite adherence to the regimen, device-assisted PFMT was introduced to enhance muscle strength. In addition, some patients reported frequent urination with small voided volumes. These patients would also receive bladder training to improve bladder capacity and reduce daytime and nighttime toileting frequency.
d. *Screening and referral for sexual dysfunction*: Recognizing that postoperative erectile dysfunction is often underreported [12], the nurse specialist routinely assessed sexual function recovery and encouraged open discussion. However, some patients felt uncomfortable to discuss this private problem that undermined their male identity. Thus, for those young patients or those indicated desire for sexuality, early referral to andrologists for evaluation and rehabilitation will be part of our regular practice. This ensured that patients received appropriate medical or psychosexual support throughout their recovery.

### Evaluation

The primary endpoint is post-operative complications, including post-operative complications and re-admission within 90 days. The secondary endpoints are the length of stay (LOS), urinary recovery, and quality of life. Post-operative complications including infection (wound infection, urinary tract infection, etc), deep venous thrombosis, bleeding, and urinary fistula. Urinary recovery was assessed by urinary leakage after the removal of urinary catheter and the ICIQ-SF questionnaire. The ICIQ-SF showed high internal consistency reliability globally across different populations [13]. It assessed the frequency, severity, and overall quality of life, ranging from 0 to 21, where higher scores indicated greater severity of incontinence. Health-related quality of life was assessed through the disease-specific instrument, the FACT-P questionnaire, which consisted of 27-item core FACT-General (covering physical, social, emotional, and functional well-being) and a 12-item prostate cancer–specific subscale (PCS). The FACT-P demonstrated strong internal consistency and reliability in prostate cancer, with higher scores reflecting better quality of life[14]. The ICIQ-SF and FACT-P were collected after the removal of urinary catheter, three months and six months after the surgery.

### Data collection and statistical analysis

The nurse specialist and nurse manager jointly designed and distributed the standardized data sheet to all team members. The dataset was shared on an online platform accessible only to authorized personnel for editing. Perioperative data were collected from the hospital’s electronic information system by the assisting physician or the research nurse. The nurse specialist was responsible for collecting ICIQ-SF and FACT-P data at the specified time points. Postoperative complications were recorded by the assisting physician and nurse specialist during the patients’ follow-up visits with the urological surgeons. Team members routinely reviewed the database and contacted patients to resolve any missing data. All data entries were independently verified by two research team members to ensure accuracy.

Quantitative data were analyzed using SPSS 22.0 (IBM Corporation, Armonk, NY, USA). Continuous variables were assessed for normality using the Shapiro-Wilk test and are presented as mean ± standard deviation (SD). Between-group comparisons at each time point were performed using independent-samples t tests, while categorical variables were compared using the chi-square test or Fisher’s exact test as appropriate. To evaluate longitudinal changes in ICIQ-SF and FACT-P scores (including subscales) measured at catheter removal, 1 month, and 3 months postoperatively, repeated-measures analysis of variance (ANOVA) was conducted to examine the main effects of time, group, and the time × group interaction. Given the large sample size, parametric methods were considered appropriate based on the robustness of ANOVA under the central limit theorem. Assumptions of residual normality and sphericity were assessed prior to model fitting. In addition to P values, between-group mean differences were reported with corresponding 95% confidence intervals (95% CIs). Standardized effect sizes (Cohen’s d) were calculated at each time point using pooled standard deviations and interpreted according to conventional thresholds (0.2 = small, 0.5 = moderate, 0.8 = large). A two-tailed P value<0.05 was considered statistically significant.

## Results

### Population demographics

Table 2 presents the baseline characteristics of the two patient groups before surgery. There were no significant differences in age, Gleason score, or comorbidities between the groups (all P > 0.05).

**Table 2:** Preoperative demographics and clinical characteristics.

Variables	Improved care model (n = 1062)	Conventional group (n = 673)	Z/χ²	P value
Age (years)	70(65,74)	69(64,74)	1.098	.272
Gleason Score	7 (6 7)	7 (6 7)		
PSA, ng/ml	10.41(6.93,17.5)	11.2(6.74,19.7)	−1.34	.179
Extent of lymph node dissection, n (%)			0.98	.611
Limited	54(5.1)	36(5.3)		
Extended	11(1)	4(0.6)		
Comorbidity, n (%)				
Hypertension	172 (16.2)	115 (17.1)	1.76	.414
Diabetes	202 (19)	105 (15.6)	3.28	.07
Heart	85(8)	62 (9.2)	3.64	.162
Cerebral infarction	77 (7.2)	54 (8)	0.36	.549

Abbreviations: PSA, prostate-specific antigen

### Postoperative outcomes

Data regarding surgical techniques and postoperative complications are summarized in Table 3. No significant differences were observed between the two groups in terms of surgical approach or lymph node dissection (P > 0.05). The incidence of postoperative complications within 30 days, despite urinary fistula, was also comparable between groups (P > 0.05). Notably, the length of hospital stay was reduced by approximately one day in the improved care model group (P < 0.001).

**Table 3:** Postoperative outcomes between two groups.

Parameters	Improved care model (n = 1062)	Conventional group (n = 673)	Z/χ²	P value
Surgical approaches, n (%)			5.5	.064
Robotic	999(94)	642(95.4)		
Laparoscopic	64(6)	29(4.3)		
Open	0	2(0.3)		
Extent of lymph node dissection, n (%)			0.98	.611
Limited	54(5.1)	36(5.3)		
Extended	11(1)	4(0.6)		
LOS, days	5 (4 6)	6 (5 6)	−12.665	<0.001
Postoperative complications within 30 days, n (%)				
Infection	70 (6.6)	49 (7.3)	0.31	.576
Deep venous thrombosis	16 (1.5)	17(2.5)	2.30	.129
Bleeding	9(0.8)	6(0.9)	0.01	.922
Urinary fistula	8 (0.8)	18 (2.7)	10.31	.001

Abbreviations: LOS, length of stay

### Rehabilitation outcomes

The scores of ICIQ-SF and FACT-P (including all subdomains) were non-normally distributed according to the Shapiro-Wilk test; therefore, data were expressed as median (interquartile range). Friedman tests revealed significant differences across the three time points (catheter removal, 1 month postoperatively, and 3 months postoperatively) in both ICIQ-SF and FACT-P scores (all P < 0.001). Post hoc Wilcoxon signed-rank tests with Bonferroni correction further confirmed that urinary symptoms and quality of life improved significantly over time.

Descriptive statistics of ICIQ-SF and FACT-P scores at different time points are presented in Table 4. Both groups showed a steady decline in ICIQ-SF scores and a progressive improvement in FACT-P scores from catheter removal to 3 months postoperatively (Table 4). Patients receiving the enhanced nursing intervention achieved significantly better functional recovery and quality-of-life outcomes compared with those receiving conventional care (P < 0.001). Repeated-measures ANOVA revealed a significant main effect of time (all P < 0.001) and a significant time x group interaction (all P < 0.001), indicating distinct improvement trajectories between groups. Regarding the FACT-P subscales, both the physical well-being and prostate cancer-specific (PCS) domains increased consistently over time, with significant time effects and time x group interactions (all P < 0.001), suggesting superior recovery trajectories in the enhanced-care group.

**Table 4:** Changes in ICIQ-SF and FACT-P Scores Over Time in Two Groups.

Variables	Removal of urinary catheter			One month after the surgery			Three months after the surgery			Time effect (F/P)	Group effect (F/P)	Interaction (F/P)
	Improved care model (n = 1062)	Conventional group (n = 673)	P value	Improved care model (n = 1062)	Conventional group (n = 673)	P value	Improved care model (n = 1062)	Conventional group (n = 673)	P value			
ICIQ-SF	6 (5,8)	8 (6,9)	<0.001	7 (5,7)	6 (5,7)	.223	6 (5,7)	6 (5,6)	.024	F = 72.93, p < 0.001	F = 77.20, p < 0.001	F = 25.59 p < 0.001
FACT-P	59(49,68)	56(47,65)	<0.001	86(77,95)	78(69,86)	<0.001	104(96,112)	94(86,102)	<0.001	F = 41729.21, p < 0.001	F = 167.67, p < 0.001	F = 328.21, p < 0.001
PWB	11 (6,16)	9 (5,13)	<0.001	17 (12,21)	14 (9,18)	<0.001	20 (17,23)	18 (14,22)	<0.001	F = 8258.63, p < 0.001	F = 107.20, p < 0.001	F = 46.12, p < 0.001
SWB	10 (6,14)	11 (7,16)	<0.001	16 (12,20)	16 (12,20)	.538	20 (17,22)	20 (15,22)	<0.001	F = 13788.92, p < 0.001	F = 0.148, p = 0.7	F = 243.87, p < 0.001
EWB	7 (5,10)	7 (5,11)	.821	11 (8,15)	11 (8,16)	.492	14 (11,18)	14 (10,18)	.016	F = 4164.42, p < 0.001	F = 0.422, p = 0.516	F = 12.80, p < 0.001
FWB	11 (6,16)	12 (8,17)	<0.001	17 (12,21)	16 (12,21)	.177	20 (16,21)	20 (15,21)	.002	F = 7120.52, p < 0.001	F = 0.26, p = 0.612	F = 144.84, p < 0.001
PCS	20 (13,27)	16 (10,21)	<0.001	25 (18,33)	20 (14,27)	<0.001	31 (24,39)	25 (20,32)	<0.001	F = 15271.21, p < 0.001	F = 178.269, p < 0.001	F = 135.18, p < 0.001

Abbreviations: ICIQ-SF: International Consultation on Incontinence Questionnaire Short Form; FACT-P: Functional Assessment of Cancer Therapy-Prostate; PWB: physical well-being; SWB: social well-being; EWB: emotional well-being; FWB: functional well-being; PCS: prostate cancer subscale.

The improved care group demonstrated significantly higher FACT-P scores at all follow-up time points (all P < 0.01). The magnitude of the between-group difference increased over time, with effect sizes of 0.26 (small) at the first assessment, 0.71 (moderate-to-large) at 1 month, and 0.89 (large) at 3 months, indicating a progressively strengthening intervention effect (Table 5). The incidence of urinary fistula was significantly lower in the improved care group (8/1055, 0.76%) compared with the conventional group (18/655, 2.75%), corresponding to a relative risk of 0.28 (95% CI 0.12–0.64, P = 0.001).

**Table 5:** Between-group comparisons of FACT-P scores.

Time point	Conventional group (Mean±SD)	Improved care (Mean±SD)	Mean Difference (95% CI)	P value	Cohen’s d
Catheter removal	55.92 ± 11.78	59.07 ± 12.56	−3.155 (−4.323--1.986)	<0.001	0.26
1 month	77.63 ± 12.07	86.45 ± 12.79	−8.822 (−10.015--7.628)	<0.001	0.71
3 months	94.04 ± 11.33	104.17 ± 11.31	−10.136 (−11.231--9.042)	<0.001	0.89

## Discussion

Established enhanced recovery protocols are available in different countries and languages, however, protocols might vary at each institution [15,16]. The improved care model in our study was developed based on current evidence and clinical practice, aiming to support the long-term survivorship and structured follow-up. This model integrated multi-disciplinary expertise to address the evolving needs of patients across the survivorship trajectory. In this study, we implemented this model to systematically evaluate its feasibility, effectiveness, and potential for broader clinical adoption.

One of the most interesting findings is the subtle fluctuation of urinary continence for patients with personalized PFMT with bladder training. Early postoperative continence recovery was associated with specialized nurse-led education, prehablitation, PFMT despite surgical- and personal- related factors [17,18]. This may explain the better ICIQ-SF outcomes observed immediately after catheter removal in our enhanced-care group, even no difference was observed with the amount of urinary leakage after catheter removal. However, the between-group difference of ICIQ-SF was no longer significant at the 1-month follow-up. The possible underlying reason is that one month after the surgery was the time that stimulated spontaneous tissue healing and neuromuscular re-adaptation [19]. Moreover, significant differences reappeared at 3 months, suggesting a sustained effect of continuous nurse specialist-led intervention. This aligns with evidence that supervised PFMT and structured survivorship support significantly improve medium-term continence outcomes [20,21]. This fluctuating pattern, with no other similar results, supports the importance of structured, ongoing follow-up rather than short-term perioperative management alone.

The most important result is a more favourable QOL results achieved across all postoperative time points after the implementation of enhanced care model. Among the subdomains, physical well-being (PWB) and PCS demonstrated significant between-group improvements at three different time points, which is consistent with the known trajectories of postoperative functional recovery [22] and symptom relief [23]. In contrast, emotional well-being (EWB) showed no significant difference between catheter removal and 1 month but with an increase by 3 months after the surgery. Past studies also found that patients suffered psychological burden including uncertainty, fear of recurrence, and adaptation to functional changes early after radical prostatectomy [24,25]. As continence improves and patients receive sustained informational and psychosocial support, emotional well-being tends to promote gradually, which may explain the delayed improvement in EWB observed at 3 months. Similar results have also been reported in targeted psychosocal interventions [26,27] that resulted in measurable emotional improvements after the surgery.

Although overall FACT-P scores improved progressively across follow-up, no significant between-group differences were observed in the social well-being (SWB) and functional well-being (FWB) domains at the 1-month time point. This indicated that social and functional recovery after radical prostatectomy typically require more time than symptom relief or physical rehabilitation. A systematic review [28] reported that men experienced social withdrawal and reduced functional until the urinary continence recovery, as showed in 6–12 weeks after the surgery [29]. The significant between-group differences re-emerged by 3 months in both SWB and FWB suggest that the enhanced-care model presented with delayed benefits of improved coping and role adjustment through information continuity, monitoring of complications, and timely referrals for sexual rehabilitation. the between-group difference in total FACT-P score at 3 months exceeded the previously reported minimal clinically important difference (MCID) of 6–8 points for the overall instrument, as defined in validation analyses of the NCCN/FACT-P Symptom Index [30]. This indicates that the observed improvement potentially reached the threshold for clinical importance from the patient perspective, in addition to achieving statistical significance. The large effect size observed at this time point further reinforces the substantive impact of the enhanced survivorship model on patient-reported quality of life. Above findings highlight the importance of psychological surveillance and the value of continued nurse-led survivorship support to address emotional needs throughout the recovery trajectory. It underscores the unique contribution of specialized nurses in maintaining engagement, reducing uncertainty, and bridging the gap between acute postoperative care and long-term survivorship needs.

Taken together, these findings suggest that postoperative recovery following radical prostatectomy is dynamic and stage-specific rather than linear. Early differences may reflect acute functional variability and immediate educational effects, whereas the temporary convergence observed at one month likely represents a transitional phase characterized by spontaneous tissue healing, neuromuscular adaptation, and adjustment to post-discharge routines. The re-emergence of significant differences at three months across continence and psychosocial domains supports the hypothesis that sustained nurse-led follow-up and structured survivorship pathways exert cumulative benefits over time. In this context, delayed improvements in social and emotional well-being are consistent with the gradual nature of social reintegration, role redefinition, and coping adjustment after major surgery.

More importantly, the enhanced care model was associated with additional clinical benefits. In our cohort, the model was linked to a shorter length of stay (LOS) without an increase in postoperative complications, suggesting that coordinated survivorship pathways may improve perioperative efficiency while maintaining patient safety. Although the absolute reduction in LOS was modest (one day), even small decreases can be meaningful in high-volume centers by potentially improving bed turnover and resource utilization. In addition, the lower incidence of urinary fistula observed in the improved-care group reflect more standardized catheter management, early symptom surveillance, and closer postoperative monitoring. These findings align with the broader movement toward enhanced recovery protocols and suggest that structured supportive care pathways facilitate safe perioperative management in settings where shorter hospitalization is increasingly pursued.

This study has several limitations. First, as a retrospective single-center analysis, the findings primarily reflect medium-term outcomes within a specific institutional context. Future multi-center studies with extended follow-up are warranted to evaluate the durability and broader generalization of the enhanced survivorship model. Second, postoperative pathological characteristics were not incorporated into the analyses. Variations in pathological risk profiles may have influenced psychological distress, functional recovery, or complication rates, potentially introducing residual confounding. Third, the before-after design inherently carries the risk of temporal confounding. Although the core surgical approach and institutional discharge policies remained stable during the study period, incremental improvements in surgical proficiency, perioperative coordination, and evolving clinical practices may have contributed to improved outcomes. Therefore, the observed benefits should be interpreted as reflecting the combined influence of the structured nursing intervention and broader secular trends rather than definitive causal effects. In addition, baseline geriatric characteristics, such as activities of daily living, cognitive status, and social support, were not systematically assessed. These factors may influence adherence to pelvic floor muscle training and psychosocial adjustment, particularly in older patients. Finally, as the study was conducted in Shanghai, where healthcare resources and survivorship support systems are relatively well developed, caution is warranted when extrapolating the findings to other healthcare settings.

Despite these limitations, the enhanced survivorship-oriented care model provides a structured, nurse-led framework that strengthens continuity of care after radical prostatectomy. By integrating systematic follow-up, timely specialty referral, psychosocial support, and targeted functional rehabilitation, the model addresses key gaps in early survivorship care. The observed clinically meaningful improvements in patient-reported outcomes and reduction in postoperative complications support its potential value in structured survivorship management, while further prospective, multi-center studies are needed to confirm its effectiveness and generalizability.

## Conclusion

This study shows that the improved care model is a safe and effective strategy for men undergoing radical prostatectomy. It shortens hospital stay, enhances postoperative quality of life, and accelerates urinary continence recovery without increasing complications. By integrating survivorship principles into routine nursing practice, the model fills a critical gap in post-discharge support and may be suitable for broader implementation across urologic centers.
